# Supplementary material for: Three-dimensional-mapping of smooth muscle morphogenesis in the vertebrate gastrointestinal tract
Source: Sci Rep. 2025 Dec 5;15:43211. doi: 10.1038/s41598-025-27324-y (PMC12680748; doi:10.1038/s41598-025-27324-y)
Supplement: Supplementary file 1 — Supplementary Information 1. [file 41598_2025_27324_MOESM1_ESM.pdf]

Legends of supplementary Figures.

Figure 1. Validation of the specificity of αSMA and γSMA antibodies. (A) Alignment of the amino acid sequences of chick αSMA and γSMA proteins. **(B)** Western blot analysis of extracts from HEK293 cell transiently transfected with constructs encoding αSMA or γSMA, each fused to a MYC tag (MYC-αSMA and MYC-γSMA). Blots were probed with anti-αSMA, anti-γSMA, anti-MYC and anti-GAPDH antibodies. GAPDH level serves as a loading control while MYC level serves as a control for transfection efficiency.

Figure 2. Analysis of αSMA and γSMA expression. (A) Representative western blot of extracts from gizzard and colon at E5 and E5.5 probed for αSMA and Revert to evaluate total protein level. **(B)** Representative western blot of extracts from gizzard and colon collected between E6 and E8, probed for αSMA. αSMA levels were normalized to Revert total protein staining. **(C)** Analysis of αSMA expression in chick GI tract at E8.5. (a) Maximum intensity Z-projections of confocal image stacks of the entire GI tract shown as heat maps. White/red colors indicate high expression levels, while dark blue indicates low expression levels. (a') representative single Z-plane confocal image. Scale bar is indicated for each panel. Abbreviations: Pre-Int, pre-umbilical intestine; Post-Int, post-umbilical intestine; co, colon. **(D)** Representative western blot showing γSMA levels in samples analyzed in (B). γSMA levels were normalized to Revert total protein staining.

A

actin, gamma-enteric smooth muscle [Gallus gallus] NP_990503 (chick gSMA)

actin, aortic smooth muscle [Gallus gallus] NP_001026400 (chick aSMA)

```
                            Identities              Gaps
                          374/377 (99%)         1/377 (0%)

chick γSMA 1    MCEEE-TTALVCDNGSGLCKAGFAGDDAPRAVFPSIVGRPRHQGVMVGMGQKDSYVGDEA  59
                MCEEE +TALVCDNGSGLCKAGFAGDDAPRAVFPSIVGRPRHQGVMVGMGQKDSYVGDEA
chick αSMA 1    MCEEEDSTALVCDNGSGLCKAGFAGDDAPRAVFPSIVGRPRHQGVMVGMGQKDSYVGDEA  60

chick γSMA 60   QSKRGILTLKYPIEHGIITNWDDMEKIWHHSFYNELRVAPEEHPTLLTEAPLNPKANREK  119
                QSKRGILTLKYPIEHGIITNWDDMEKIWHHSFYNELRVAPEEHPTLLTEAPLNPKANREK
chick αSMA 61   QSKRGILTLKYPIEHGIITNWDDMEKIWHHSFYNELRVAPEEHPTLLTEAPLNPKANREK  120

chick γSMA 120  MTQIMFETFNVPAMYVAIQAVLSLYASGRTTGIVLDSGDGVTHNVPIYEGYALPHAIMRL  179
                MTQIMFETFNVPAMYVAIQAVLSLYASGRTTGIVLDSGDGVTHNVPIYEGYALPHAIMRL
chick αSMA 121  MTQIMFETFNVPAMYVAIQAVLSLYASGRTTGIVLDSGDGVTHNVPIYEGYALPHAIMRL  180

chick γSMA 180  DLAGRDLTDYLMKILTERGYSFVTTAEREIVRDIKEKLCYVALDFENEMATAASSSSLEK  239
                DLAGRDLTDYLMKILTERGYSFVTTAEREIVRDIKEKLCYVALDFENEMATAASSSSLEK
chick αSMA 181  DLAGRDLTDYLMKILTERGYSFVTTAEREIVRDIKEKLCYVALDFENEMATAASSSSLEK  240

chick γSMA 240  SYELPDGQVITIGNERFRCPETLFQPSFIGMESAGIHETTYNSIMKCDIDIRKDLYANNV  299
                SYELPDGQVITIGNERFRCPETLFQPSFIGMESAGIHETTYNSIMKCDIDIRKDLYANNV
chick αSMA 241  SYELPDGQVITIGNERFRCPETLFQPSFIGMESAGIHETTYNSIMKCDIDIRKDLYANNV  300

chick γSMA 300  LSGGTTMYPGIADRMQKEITALAPSTMKIKIIAPPERKYSVWIGGSILASLSTFQQMWIS  359
                LSGGTTMYPGIADRMQKEITALAPSTMKIKIIAPPERKYSVWIGGSILASLSTFQQMWIS
chick αSMA 301  LSGGTTMYPGIADRMQKEITALAPSTMKIKIIAPPERKYSVWIGGSILASLSTFQQMWIS  360

chick γSMA 360  KPEYDEAGPSIVHRKCF  376
                K EYDEAGPSIVHRKCF
chick αSMA 361  KQEYDEAGPSIVHRKCF  377
```

B

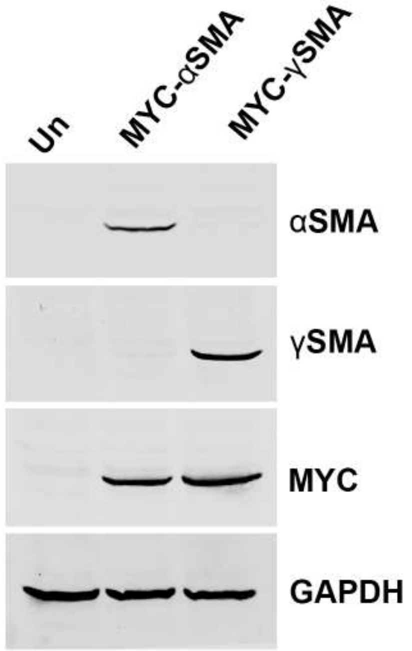

Supplementary FIG. 1

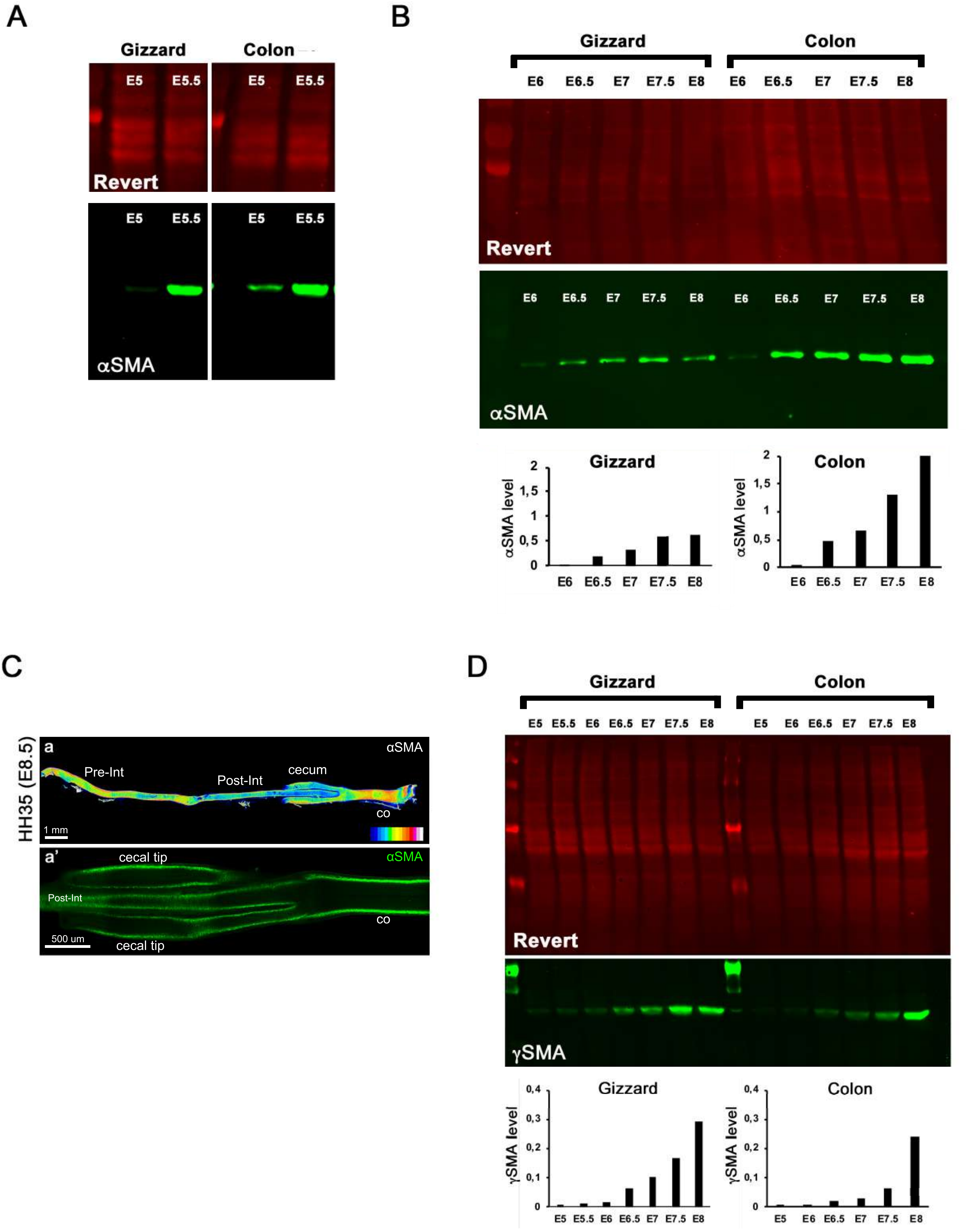

Supplementary FIG. 2
