# Supplementary material for: Three-dimensional-mapping of smooth muscle morphogenesis in the vertebrate gastrointestinal tract
Source: Sci Rep. 2025 Dec 5;15:43211. doi: 10.1038/s41598-025-27324-y (PMC12680748; doi:10.1038/s41598-025-27324-y)
Supplement: Supplementary file 2 — Supplementary Information 2. [file 41598_2025_27324_MOESM2_ESM.pdf]

Supplementary Information file

Supplementary Figure 1B

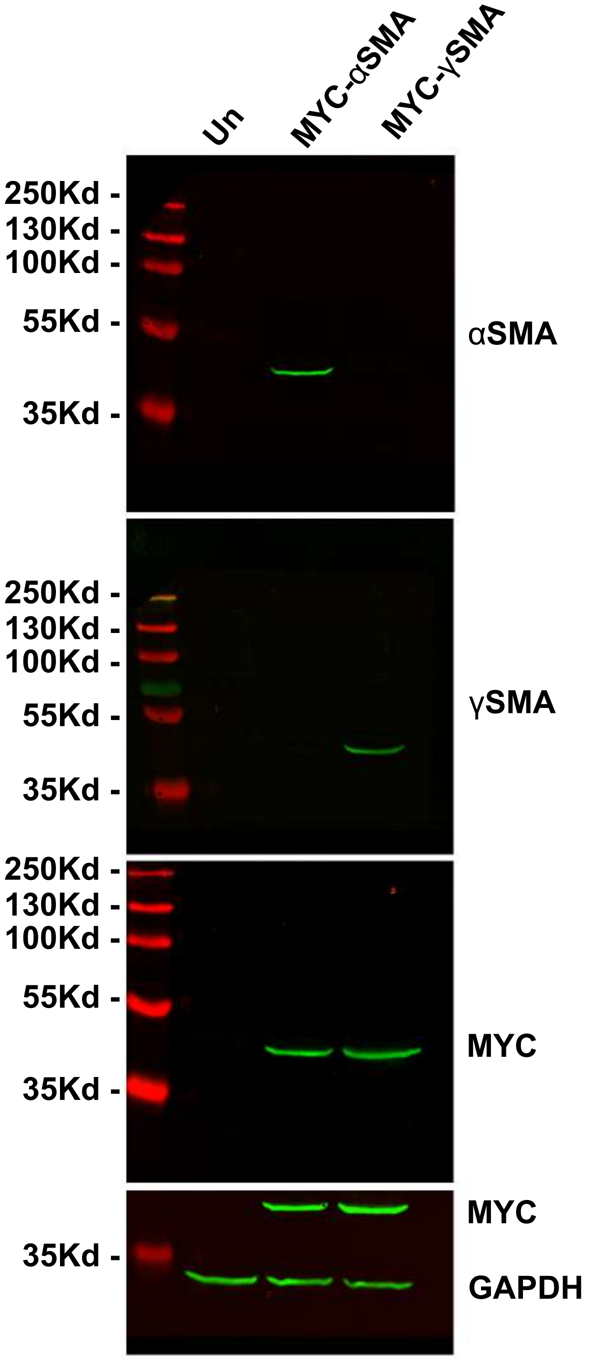

Validation of the specificity of αSMA and γSMA antibodies. Western blot analysis of extracts from HEK293 cell transiently transfected with constructs encoding αSMA or γSMA, each fused to a MYC tag (MYC-αSMA and MYC-γSMA). Blots were probed with anti-αSMA, anti-γSMA, anti-MYC and anti-GAPDH antibodies. GAPDH level serves as a loading control. MYC level serves as a control for transfection efficiency.

Supplementary Information file

Supplementary Figure 2A

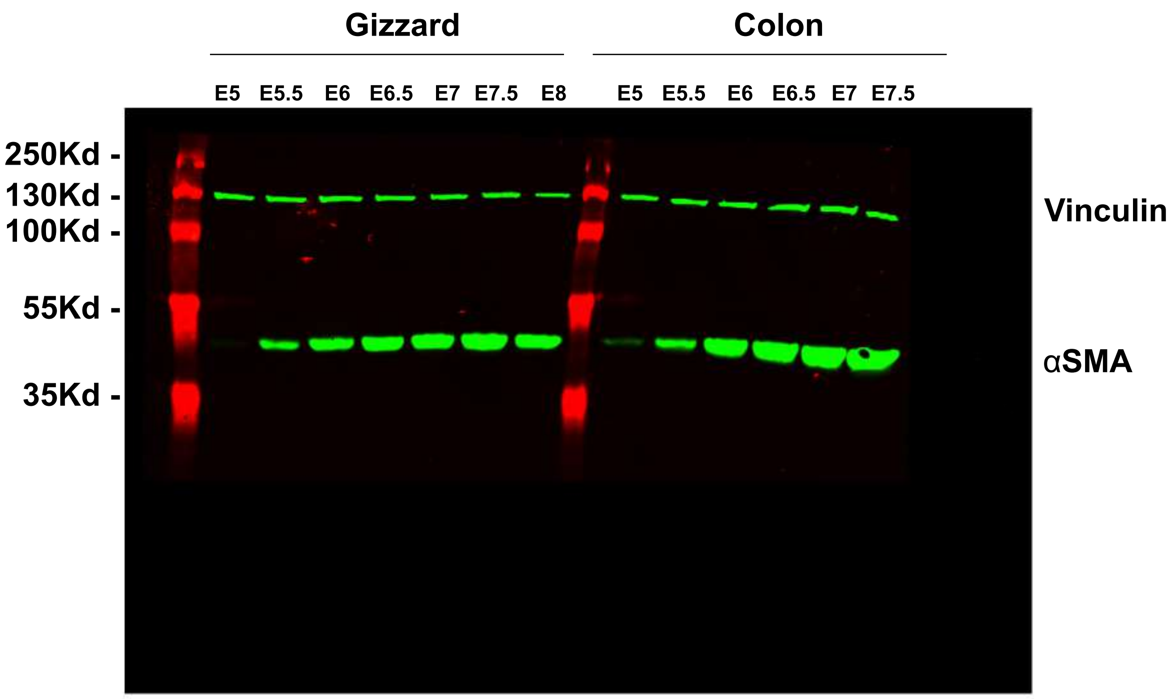

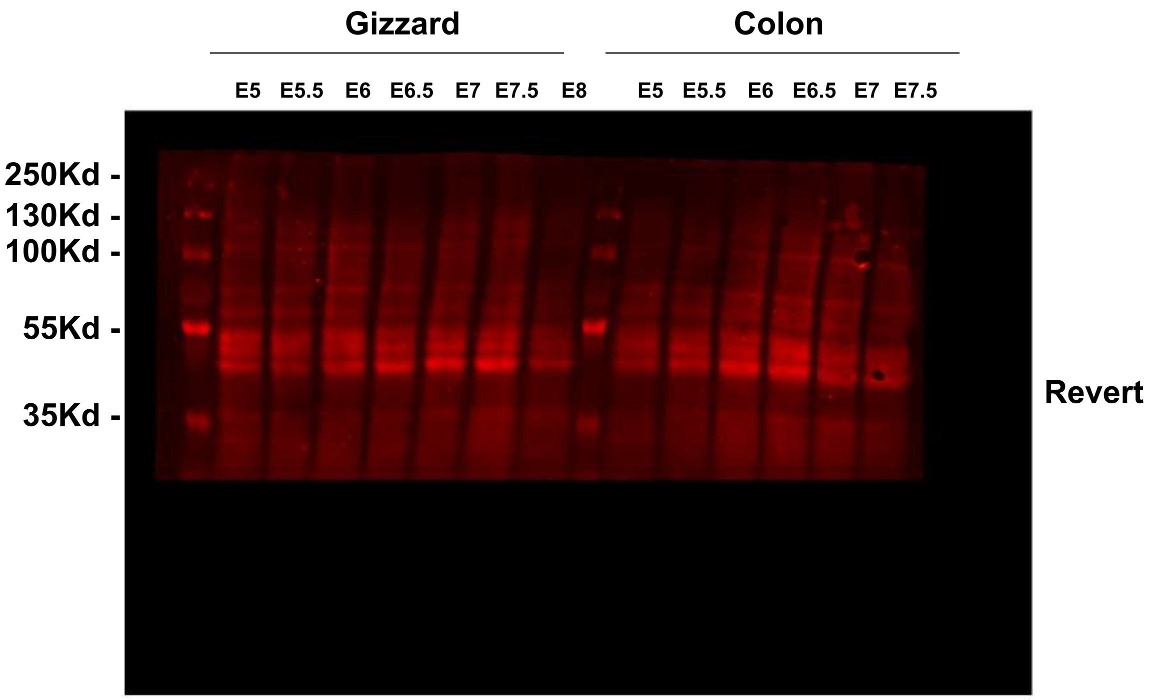

Analysis of αSMA expression. Representative Western blot of gizzard and colon extracts from E5 to E8. The blot was probed with anti-αSMA and anti-Vinculin (anti-Vinculin rabbit mAb E1E9, Cell Signaling RRID:AB_2728768) antibodies. Revert and Vinculin signals were used to assess total protein levels.

Supplementary Information file

Supplementary Figure 2B

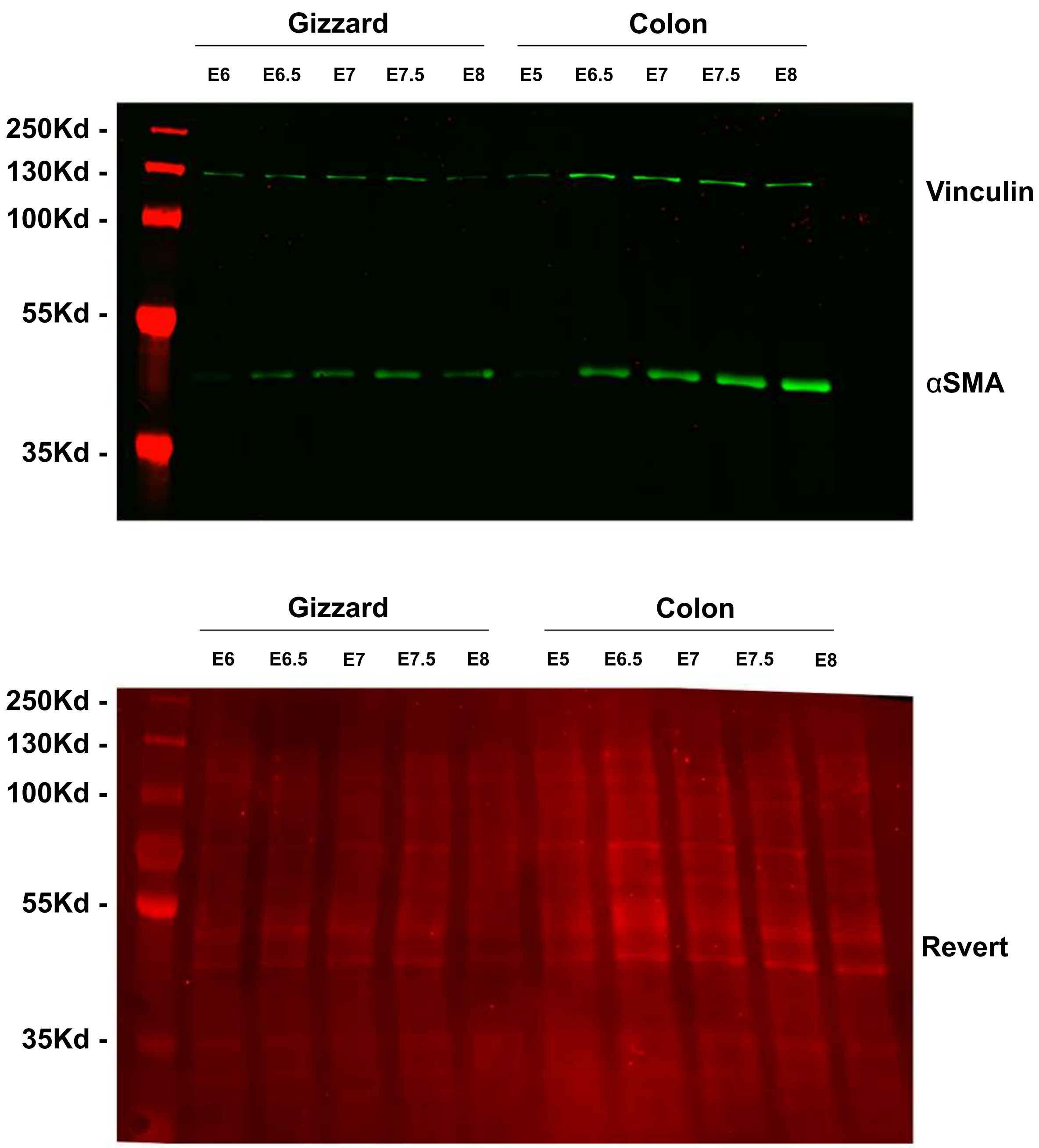

Analysis of αSMA expression. Representative western blot of extracts from gizzard and colon collected between E6 and E8. The blot was probed with anti-αSMA and anti-Vinculin (anti-Vinculin rabbit mAb E1E9, Cell Signaling RRID:AB_2728768) antibodies. Revert and Vinculin signals were used to assess total protein levels.

Supplementary Information file

Supplementary Figure 2D

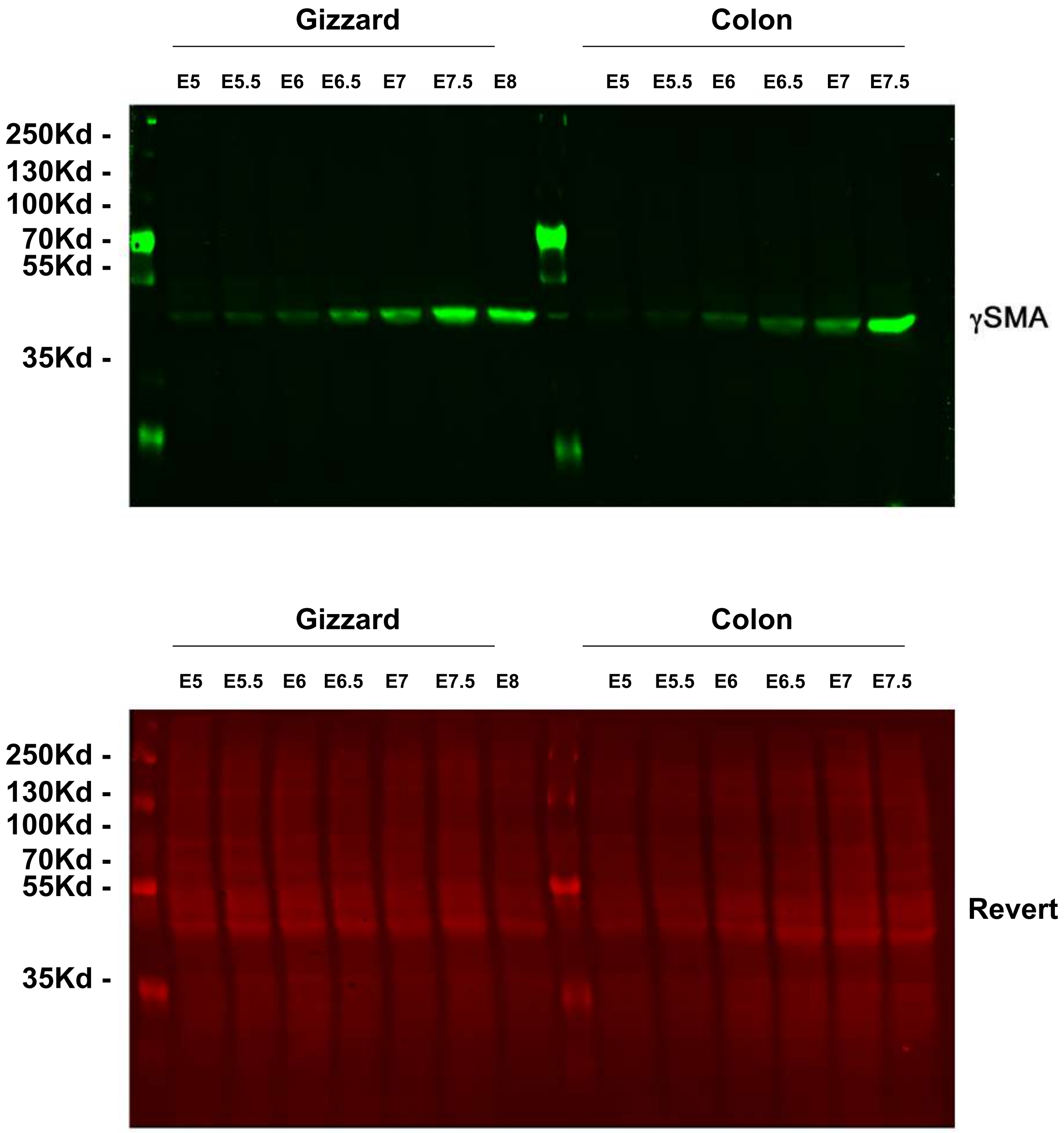

Analysis of γSMA expression. Representative western blot of extracts from gizzard and colon collected between E5 and E8. The blot was probed with anti-γSMA antibodies. Revert was used to assess total protein levels.
